# Evaluating the Role of Wnt Signal Transduction in Promoting the Development of the Heart

**DOI:** 10.1100/tsw.2007.71

**Published:** 2007-02-02

**Authors:** Leonard M. Eisenberg, Carol A. Eisenberg

**Affiliations:** Department of Cell Biology and Anatomy, Medical University of South Carolina, Charleston, USA

**Keywords:** Wnt, Dickkopf, frizzled-related proteins, cardiac development, heart, mesoderm, myocardium

## Abstract

Wnts are a family of secreted signaling proteins that are encoded by 19 distinct genes in the vertebrate genome. These molecules initiate several signal transduction pathways: the canonical Wnt, Wnt/Ca2+, and Wnt/planar cell polarity pathways. Wnt proteins have major impact on embryonic development, tumor progression, and stem cell differentiation. Wnt signal transduction also influences the formation of the heart, yet many issues concerning the involvement of Wnt regulation in initiating cardiac development remain unresolved. In this review, we will examine the published record to discern (a) what has been shown by experimental studies on the participation of Wnt signaling in cardiogenesis, and (b) what are the important questions that need to be addressed to understand the importance and function of Wnt signal transduction in facilitating the development of the heart.

## INTRODUCTION

The development of the vertebrate heart commences at the stage during embryogenesis when the three primitive germ layers (ectoderm, mesoderm, and endoderm) are first formed. Within the anterior half of the embryo, newly gastrulated mesodermal cells that have spread laterally from the primitive streak coalesce into paired heart-forming fields. As development proceeds, these bilateral distributed precardiac fields merge at the midline to give rise to the primary heart tube[1,2].

At the time the paired heart-forming mesodermal fields can be first identified by fate mapping[3,4,5], the cellular constituents of these regions are nondifferentiated progenitor cells[2,6]. Subsequently, the heart-forming fields begin to express various cardiac-associated transcription factors, such as Nkx2.5, Tbx5, MEF2c, and GATA6[3,7,8,9,10,11], and then, as the paired fields begin to fuse, myofibrillar proteins[12,13,14,15,16,17]. Although the precardiac mesoderm gives rise to both the myocardial and endocardial layers of the primary heart tube[5,18,19], most studies examining the stimulation of heart formation, except for a few exceptions[20,21], have focused on the formation of the myocardium. The reason for this emphasis is twofold: myocytes comprise the far majority of cells within the primary heart tube and cardiomyocytes possess a phenotype that is both unique to the heart and defines the function of this organ. Accordingly, this review on heart formation is, more accurately, a discourse on myocardial development.

Multiple secreted signaling molecules have been implicated as primary stimuli of cardiogenesis including insulin-like growth factors (IGF), transforming growth factor (TGF)-α, cripto, fibroblast growth factors (FGFs), and bone morphogenetic proteins (BMPs)[22,23,24,25,26,27]. In recent years, Wnt signaling has been accorded great attention for its putative importance as an essential regulator of heart formation[28,29,30,31]. Wnts, Wnt receptors, and Wnt inhibitors display dynamic temporal and spatial patterns in conjunction with precardiac tissue and within the developing heart[32]. The expression data would appear to suggest that Wnt signaling plays a multifaceted role in the development of the heart, yet most of the focus on the importance of Wnt proteins for cardiac development has been on their perceived effect on the initiation of cardiogenesis. It is hypothesized, and readily accepted, that antagonism of "canonical" Wnt signaling is a necessary precondition for heart formation and the display of a cardiomyocyte phenotype[29,31,33,34,35,36]. Here, we will scrutinize this hypothesis and examine whether it is supported by the experimental record. To be effective in this critique, we will adopt the tone of devil's advocate, in order to shed light on the role that Wnt signaling may actually play in heart development. No study, including our own, will be examined with less than a very critical eye. But first, we begin by describing the signaling pathways that are triggered by Wnt proteins.

## WNT PROTEINS AND THEIR SIGNALING PATHWAYS

Wnts are a large family of secreted signaling proteins encoded by 19 distinct genes in the mammalian genome. Wnt functional activity has been well characterized for its major influence on embryonic development, tumor progression, and stem cell differentiation[37,38,39]. The molecular biology underlying Wnt functional activity has proven to be rather complex, as it involves multiple cell membrane receptors, multiple classes of extracellular Wnt regulatory proteins, and multiple signal transduction pathways. The overview provided below of the molecular biology of Wnt signaling will reckon with these complexities, although only in sufficient detail for understanding the signal transduction issues that are relevant to a discussion of cardiac developmental biology. For a more comprehensive description of Wnt signal transduction, there are many excellent reviews and essays that can be recommended[37,40,41,42,43]. A useful resource for keeping abreast of the latest findings on this topic is "The Wnt Homepage" at http://www.stanford.edu/~rnusse/wntwindow.html.

The past several years have seen a considerable evolution in the conception of Wnt signal transduction. Wnt functional activity was thought to result solely from an individual signal transduction pathway (Fig. 1), which is now referred to as the canonical Wnt pathway[37,43,44]. This pathway is triggered when Wnt proteins bind to their cell membrane receptors, which are the ten known members of the frizzled family of transmembrane proteins[37,40,45]. Transduced signal from this ligand/receptor interaction — relayed through the cytoplasmic protein Dishevelled (Dsh)[46] — promotes the inactivation of glycogen synthase kinase 3 (GSK3). In the absence of Wnt protein, GSK3 phosphorylation of β-catenin targets the latter protein for ubiquitin degradation. The inhibition of GSK3 activity by the Wnt signal results in the cellular accumulation of β-catenin, which then translocates to the nucleus and forms a transcriptional enhancer complex with LEF/TCF DNA binding proteins[37,43,44]. Other key molecules in the canonical Wnt pathway include the low-density lipoprotein receptor-related protein (LRP) isoforms LRP5 and LRP6, which function as coreceptors for Wnt binding to the cell membrane[47], and Axin, which facilitates GSK3 phosphorylation of β-catenin by forming a cytoplasmic complex with these other proteins[48]. Wnt binding to LRP5/6 helps to dissociate the β-catenin destruction complex by redistributing Axin within the cell, thereby assisting Wnt inhibition of GSK3 activity[49].

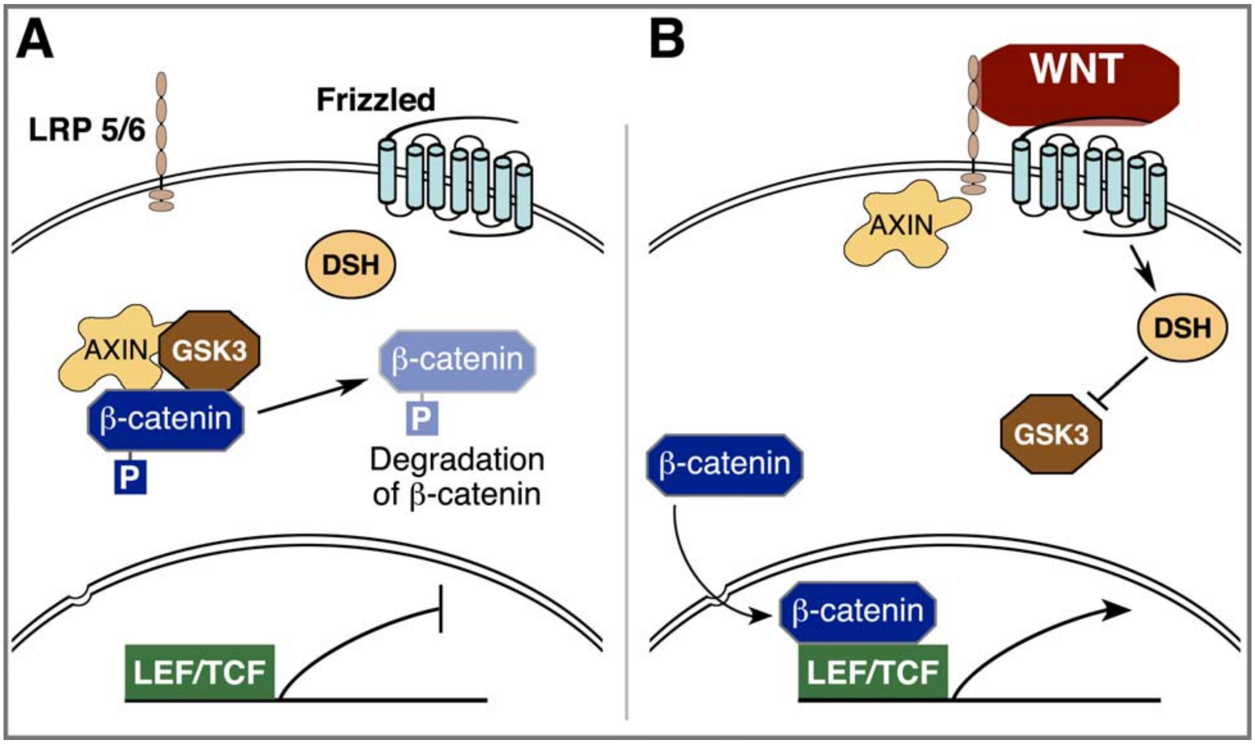

**FIGURE 1**. The canonical Wnt signal transduction pathway. (A) In the absence of Wnt protein, β-catenin is sequestered within a multiprotein cluster that includes glycogen synthase kinase 3 (GSK3) and Axin. GSK3 phosphorylation of β-catenin targets the latter protein for degradation by ubiquination. (B) Wnt binding to frizzled and LRP5/6 transmembrane proteins transduces signal via the cytoplasmic protein Dishevelled (Dsh) and causes the inactivation of GSK3. This action preventing GSK3 tagging of β-catenin allows the latter protein to accumulate within the cytoplasm. Subsequently, β-catenin translocates to the nucleus and forms a transcriptional enhancer complex with LEF/TCF DNA binding proteins, which results in the up-regulation of Wnt target genes. More detailed information on canonical WNT signal transduction can be found at "The Wnt Homepage" at http://www.stanford.edu/~rnusse/wntwindow.html.

The existence of multiple of Wnt signaling pathways is now readily accepted. In contrast to the canonical Wnt pathway, the available data do not yet allow for a straightforward description of the alternative noncanonical Wnt signaling pathway(s). Because the identification of noncanonical Wnt signaling is relatively recent, many molecular details have not been elucidated. To date, a number of molecules have been implicated in noncanonical Wnt signaling (Fig. 2) including G-proteins, the transmembrane protein strabismus, phospholipase C (PLC), protein kinase C (PKC), Ca2+/calmodulin-dependent protein kinase II (CaMKII), c-jun kinase (JNK), Rho family GTPases, and intracellular Ca2+ release[41,50,51,52,53,54,55]. Noncanonical Wnt signaling is often described as representing two distinct pathways, which are the Wnt/Ca2+ and Wnt/planar cell polarity (PCP) pathways[41,50,56,57]. However, plausible models that incorporate these disparate components into a single pathway or Wnt regulatory network have also been proposed[55,58].

An additional level of complexity to Wnt signaling relates to the divergence of functional activities among members of this protein family. This diversity has led to the classification of Wnts into two separate groups[59,60]. The Wnt1 group, which includes Wnt1, Wnt3A, and Wnt8, appears to transduce signal exclusively via the canonical Wnt/β-catenin pathway[37]. In contrast, the Wnt5A group, which consists of Wnt4, Wnt5A, and Wnt11, possesses more elaborate signaling properties. In most instances where these latter molecules have been examined experimentally, their functional activities proceeded from noncanonical Wnt signal transduction[59,61,62,63,64]. Moreover, noncanonical Wnt signaling instigated by the Wnt5A group (including Wnt11) may suppress β-catenin–mediated signaling (i.e., canonical Wnt signaling)[65,66,67,68,69]. In other words, this subgroup of Wnt proteins can serve as dominant-negative inhibitors of Wnt1 class proteins (Fig. 3). As a further complication, specific Frizzled isoforms can alter

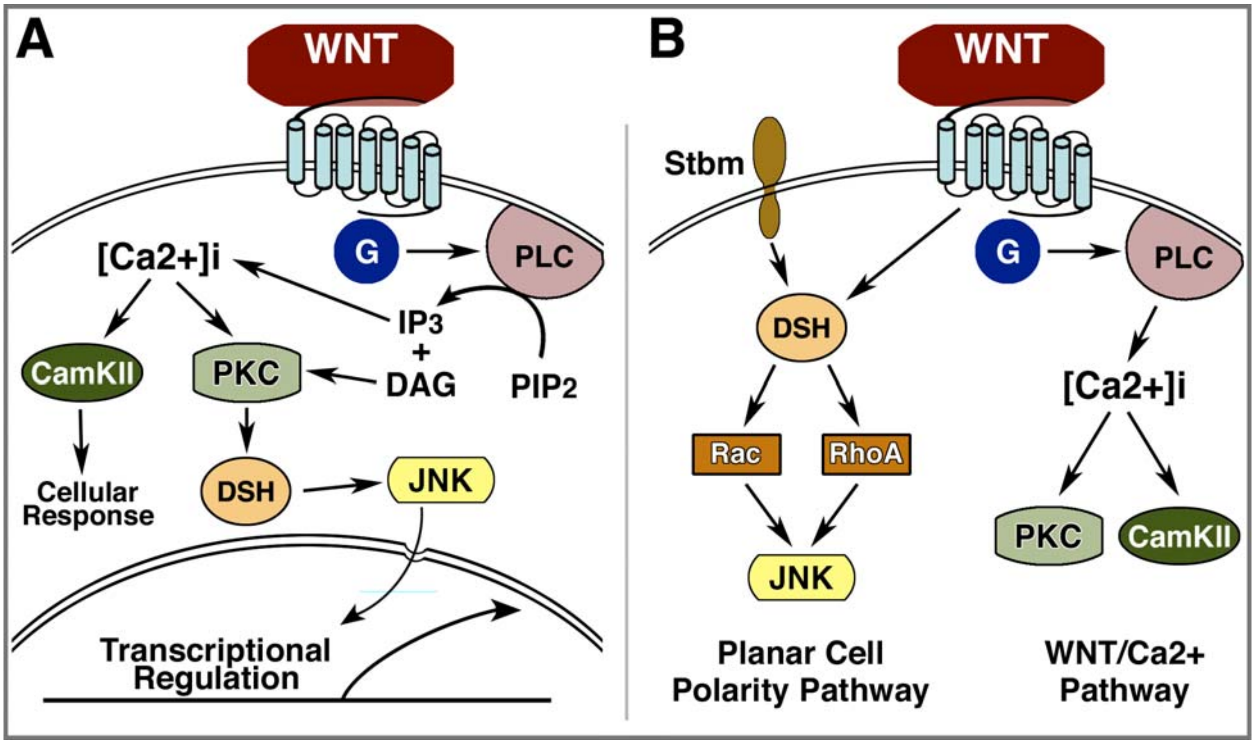

**FIGURE 2.** Noncanonical WNT signal transduction pathway(s). (A) Depiction of a single noncanonical Wnt pathway involving G-proteins, phospholipase C (PLC), protein kinase C (PKC), c-jun kinase (JNK), Ca2+/calmodulin-dependent protein kinase II (CaMKII), and Dsh. Key features include the hydrolysis of phosphatidylinositol bisphosphate (PIP2) by PLC to produce diacylglycerol (DAG) and inositol triphosphate (IP3), which in turn promotes intracellular Ca2+ release. Activated PKC regulates Dsh protein, which subsequently stimulates JNK activity. (B) Noncanonical Wnt signaling is often portrayed as two distinct pathways, which are the planar cell polarity (PCP) and Wnt/Ca2+ pathways. Molecules that may distinguish the PCP pathway are the transmembrane protein strabismus (stbm), which is a putative regulator of Dsh; and the Rho family GTPases, Rac and RhoA, which stimulate JNK activity. Note the involvement of Dsh in both canonical and noncanonical Wnt pathways, which may be due to altering its activities by differential phosphorylation[46]. Please refer to the text for references that provide a more in depth description of these signal transduction events.

the signaling properties of the Wnt5A group from being inhibitors into activators of the β-catenin pathway[70,71,72].

There are several types of soluble extracellular factors that inhibit canonical Wnt signaling (Fig. 3). Secreted frizzled-related proteins (sFRP) are soluble molecules that contain a region homologous to the Wnt binding domain of the frizzled cell membrane receptor proteins[73,74,75]. Thus, sFRP proteins prevent the triggering of canonical Wnt signaling by competitively inhibiting Wnt/frizzled binding. An additional means sFRPs may employ to inhibit Wnt activity is to form heteromers with frizzled proteins[76]. Six distinct sFRP genes have been discovered in the mammalian genome including sFRP3 (also known as Frzb1) and the molecule referred to as crescent[34,77,78]. Dickkopf (Dkk) proteins comprise a family of four related molecules that have been designated as canonical Wnt inhibitors[74,79,80]. Similar to Wnts, Dkk proteins bind to the cell membrane receptors LRP5 and LRP6[81,82]. Although the competitive binding of Dkk to the extracellular domain of LRP5 and LRP6 may serve as a mechanism for inhibiting canonical Wnt signaling, there is evidence that signal transduction events initiated by Dkk interactions with LRP5 or LRP6 can also play a role in their regulation of Wnt activity[83]. In addition, the influence of Dkk proteins on Wnt signaling may be mediated through their interaction with the Kremen transmembrane proteins[84,85].

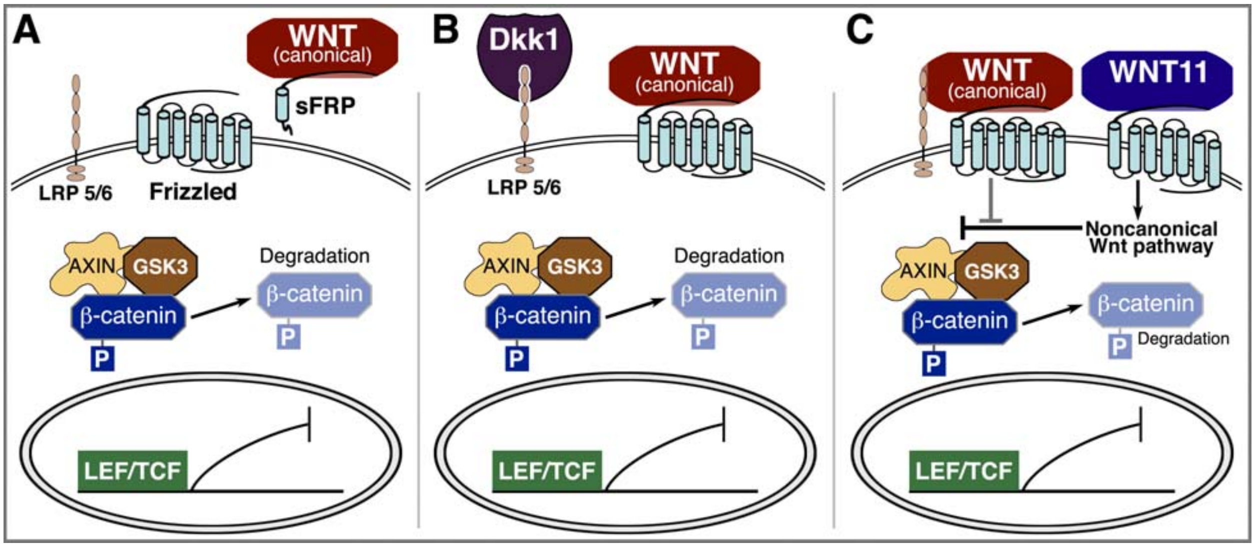

**FIGURE 3.** Multiple types of secreted, extracellular factors block canonical Wnt signal transduction. (A) sFRPs prevent Wnt binding to its cell membrane receptor frizzled by its competitive association with Wnt proteins. (B) Dkk1 interferes with the canonical Wnt pathway by binding to LRP5/6, which prevents Wnt proteins from interacting with this second class of Wnt receptors. (C) Noncanonical Wnt proteins (e.g., Wnt11) can inhibit the canonical Wnt pathway by downstream signal transduction events. Although the molecular details accounting for this suppression have not been well characterized, the activity of noncanonical Wnt signaling is known to prevent GSK3 inactivation by canonical Wnts.

## INITIATION OF HEART FORMATION — THE CANONICAL WNT INHIBITION HYPOTHESIS

The current paradigm on Wnt signal transduction and cardiac development is that inhibition of canonical Wnt signaling initiates cardiogenesis. Specifically, it is hypothesized that diffusion of Wnt antagonists induce heart formation in primary lateral mesoderm by establishing a zone of low canonical Wnt activity[34,35]. Studies in both the embryonic frog and bird (chick and quail) have identified three distinct antagonists of the canonical Wnt pathway (Dkk1, crescent, and Wnt11) as mediators of cardiac induction[34,35,62,86]. The question to be posed here is whether, in actuality, the published record is supportive of this hypothesis.

Studies in the embryonic frog have loomed large in codifying current thinking about the role of Wnt signal transduction in cardiac development. In the frog, paired heart-forming fields arise from mesoderm lateral to Spemann's Organizer at the dorsal marginal zone (DMZ). In contrast, mesoderm tissue within the ventral marginal zone (VMZ) does not provide cellular material to the developing heart[87,88,89]. Experiments investigating the induction of cardiac tissue formation from VMZ explants or suppression of cardiogenesis in DMZ explants have provided important information on the inductive events underlying the formation of the frog heart[35,62]. The functional role of individual genes in heart development has been examined by injecting RNA into individual blastomeres at the two- or four-cell stage (Fig. 4). The injection into both ventral blastomeres of a four-cell stage embryo of mRNA encoding for Dkk1 and crescent — molecules viewed strictly as Wnt inhibitors — allowed VMZ explants to generate cardiac tissue[35]. Similarly, injection of canonical Wnts (Wnt3A and Wnt8A) into the dorsal blastomeres at the four-cell stage decreased by 50% the number of DMZ explants that displayed cardiac tissue[35]. In a subsequent study, the ventral injection of Wnt11 RNA was shown to also stimulate the formation of heart tissue from VMZ explants[62]. Furthermore, this latter study showed evidence that cardiac induction by Wnt11, Dkk1, and crescent was mediated through activation of Jun N-terminal kinase (JNK), which has been characterized as a component of noncanonical Wnt signaling (Fig. 2). These data suggest that not only Wnt11, but also Dkk1 and crescent, can initiate signal transduction events ascribed to the noncanonical Wnt pathway.

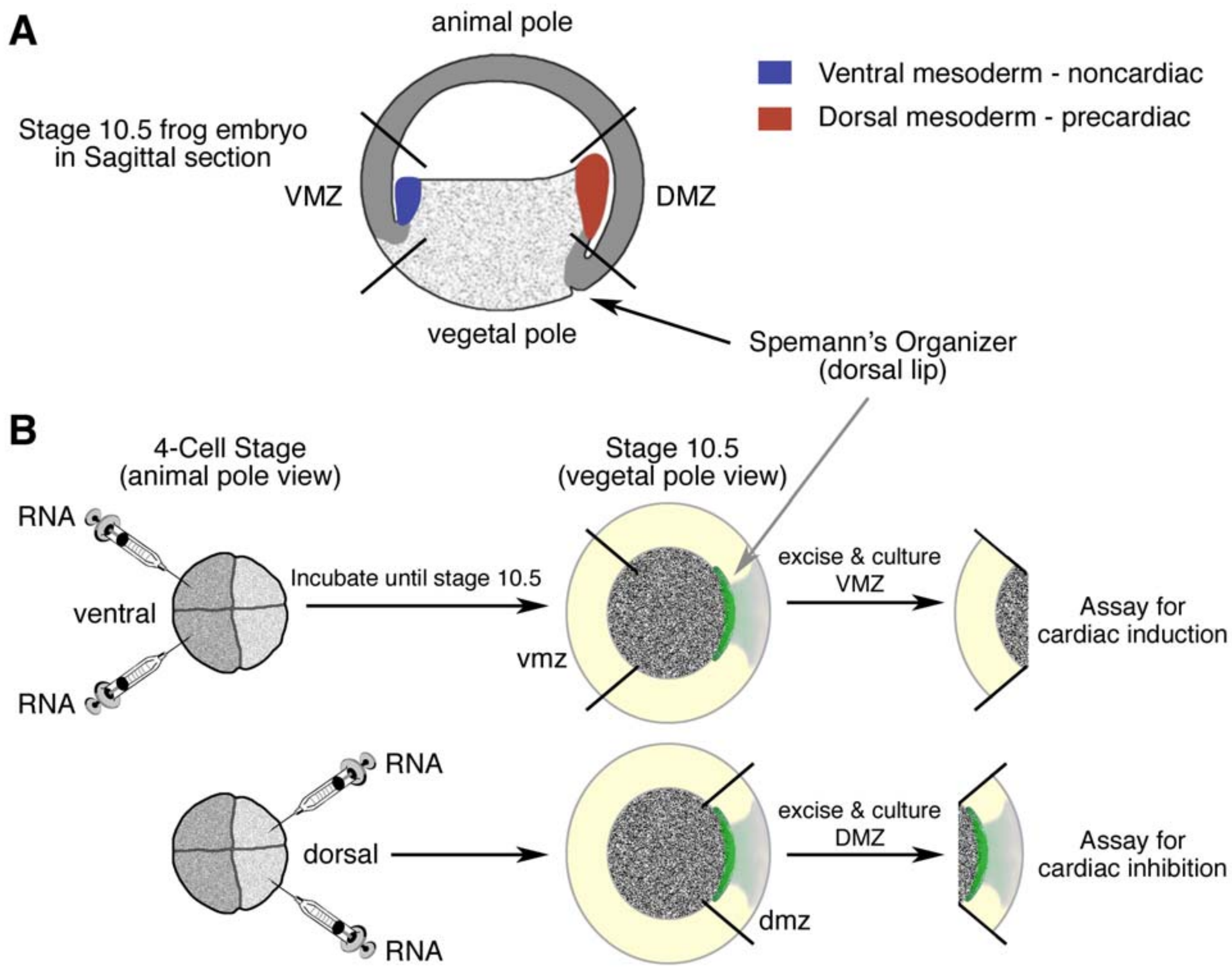

**FIGURE 4.** Cardiac tissue formation in *Xenopus* tissue explants. (A) Drawing depicts an early frog gastrula (stage 10.5) in sagittal section. Highlighted are the dorsal mesoderm (red), which contains the bilaterally distributed precardiac tissue fields, and the ventral mesoderm (blue), which does not contribute cells to the heart *in situ*. (B) Experimental outline used to study the regulation of heart formation in the frog. The functional role of individual genes in heart development is examined by injecting their RNA into either the ventral or dorsal blastomeres at the two- or four-cell stage. After allowing the embryos to develop to stage 10.5, the ventral marginal zone (VMZ) or dorsal marginal zone (DMZ) is excised and incubated in culture. Subsequently, these explants are examined for cardiac gene expression and the formation of a contractile phenotype. Experiments investigating the induction of cardiac tissue formation from VMZ explants or suppression of cardiogenesis in DMZ explants have provided important information on the regulatory events underlying the formation of the frog heart.

The published reports of ectopic cardiac induction in VMZ tissue was judged by a recent study to be an artifact due to overexpression of the microinjected molecules[90]. The specific argument was put forward that Wnt11 stimulation of heart formation in the frog may be spurious due to the high amount (1 ng) of Wnt11 RNA used for the injections. However, a number of counterarguments can be offered to offset this objection. First, the only study directly comparing the cardiogenic activities of Dkk1, crescent, and Wnt11[62] used 0.8, 0.9, and 1.0 ng of RNA, respectively, to maximize expression of cardiac marker genes by VMZ tissue. Those concentrations are hardly statistically significant and reflect a near-equivalent ability of the three molecules to stimulate cardiac gene expression. Moreover, in the study that first established the putative role of Wnt antagonists to promote cardiogenesis, 1.5 ng of crescent RNA per injection was needed to elicit a contractile phenotype[35]. Thus, if the case against the cardiac induction data in the frog embryo has any validity, it extends to each of the candidate inducers of cardiogenesis: Wnt11, Dkk1, and crescent.

Second, RNA concentrations of 1 ng or higher are very commonly used in microinjection studies in the frog embryo. To illustrate this point, we scanned through articles published in one journal during a single year (*Developmental Biology,* 2005) and found a multitude of studies from many of the world's top frog development laboratories that used 1 ng or higher RNA concentrations for microinjecting *Xenopus* embryos[91,92,93,94,95,96,97]. Therefore, if the cardiac-inducing microinjection studies are to be dismissed as being artifact, then that rejection must extend to innumerous studies unrelated to heart formation and represents a critique of one of the fundamental methods used to study frog development. It is beyond the scope of this essay to refute that argument on these grounds.

Third, the knockdown of Wnt11 expression by injection of gene-specific morpholino antisense oligonucleotides dampened both cardiac gene expression and interfered with normal cardiac development[62]. Despite the absence of similar data for Dkk1 and crescent, the published data at least provide evidence that Wnt11 expression does have a role in promoting heart formation. Although one would like to see this experiment repeated by another laboratory in order to codify the finding, these data on the inhibition of endogenous Wnt11 expression are more difficult to dismiss than the ectopic expression studies.

There is, however, another and more valid critique to be made on the interpretation of the RNA microinjection studies. The term that is often bandied about to describe the importance of Wnt11, Dkk1, and crescent is that they "induce" heart formation. Induction implies a direct causative agent of cardiac differentiation by acting directly on the progenitor cells that will give rise to the heart. Yet a considerable amount of development occurs from the time RNA is administered at the four-cell stage to early gastrulation when nondifferentiated mesoderm begins to coalesce into heart-forming regions. Accordingly, the microinjection experiments cannot provide evidence that the effect of Wnt11, Dkk1, and crescent on heart development is inductive. This was conceded in a more recent study suggesting that Dkk1 and crescent actually act on the underlying endoderm to provoke the secretion of an unknown heart inducing factor[98], but that study was also based on early-stage microinjections (16- and 32-cell stages). As the timeline between the putative action of Dkk1 and crescent on endoderm and their downstream effects on precardiac cells is undefined, the functional role of these two molecules in promoting cardiogenesis remains unclear.

Experiments designed to assess the impact of Wnt11, Dkk1, and crescent on nondifferentiated mesoderm have, to date, only been performed with avian tissue (Fig. 5). Studies have examined the inductive properties of these molecules using explants of chick or quail posterior lateral mesoderm (PLM), which does not provide cells to the developing heart *in situ*. Typically, this tissue is harvested from Hamburger and Hamilton (HH) stage 5, which is the point in development when the primitive streak begins to recede and the notochord first starts to form[99]. In order to ensure survival of the explanted tissue, the mesoderm must be cultured along with the underlying endoderm (mesendoderm), or in the presence of high concentrations of serum or chick embryo extract[25,34,86,100,101,102]. As reported by our laboratory and others[34,86,101], treatment of PLM with Wnt11, Dkk1, and crescent enhanced both cardiac gene expression and formation of small cardiomyocyte-positive areas. Although these data may appear to demonstrate an inductive role of these molecules in provoking cardiogenesis, there are a number of caveats that can be made about these data. First, neither we nor others have demonstrated that the cardiac differentiation of anterior lateral (i.e., precardiac) mesoderm can be perturbed by inhibiting the expression or activity of Wnt11, Dkk1, or crescent. Second, the cardiac areas within PLM explants resulting from these treatments are limited in size as compared to the huge mounds of cardiac tissue that form from similarly staged nontreated precardiac mesoderm explants[86,101]. Of course, that may just mean other factors are involved in order to provoke full-fledged cardiac tissue formation. Yet this objection becomes more compelling in conjunction with a third observation that normally noncardiogenic PLM tissue, removed from its embryonic environment and placed in culture under nontreated and minimal media conditions, will demonstrate a limited ability to produce small areas of cardiomyocytes[86,101]. We observed that treatments with Wnt11 and Dkk1 increased the frequency of PLM explants that exhibited cardiomyocytes, but that the resulting small areas of cardiac tissue were neither larger nor of different quality than those that spontaneously arose from nontreated PLM explants.

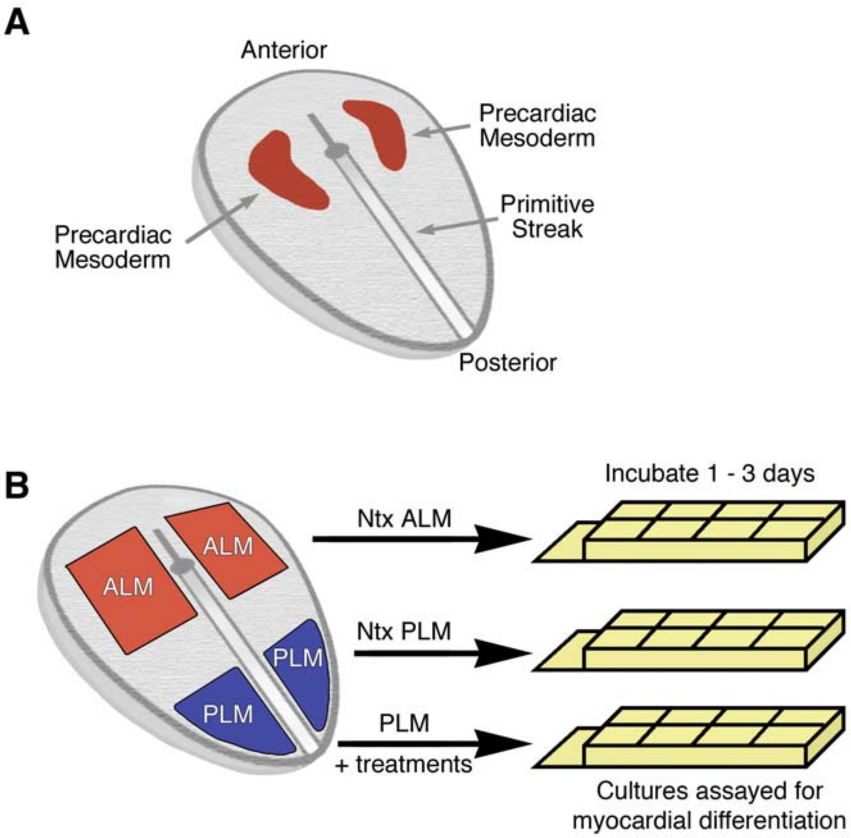

**FIGURE 5**. Cardiac tissue formation in explants of avian tissue. (A) Representation of a HH stage 5 chick embryo shown from the dorsal side, with the location of the bilateral precardiac mesodermal fields depicted in red. At this stage of development, precardiac areas are comprised of nondifferentiated cells that have not begun to express cardiac-associated markers. (B) The positions of anterior and posterior lateral mesoderm (ALM and PLM) regions used as a source of tissue explants are illustrated in this diagram of an HH stage 5 chick embryo. ALM, but not PLM tissue, will spontaneously form contractile tissue when incubated in culture. The PLM tissue, which does not contribute cells to the heart *in situ*, is commonly used as a source of noncardiac mesoderm in cardiac induction studies.

Our recent study on the phenotypic plasticity of PLM tissue indicated that Dkk1 treatments boosted the incidence of cardiac tissue formation by twofold[101]. While these latter data indicate that Dkk1 (and Wnt11) has a positive influence on the ability of mesodermal cells to exhibit a cardiac phenotype, it is not clear from these experiments whether the mode of regulation is inductive.

## IS CANONICAL WNT SIGNALING ANTITHETICAL TO THE FORMATION OF THE HEART?

As discussed in the previous section, there is evidence suggesting that Wnt11, Dkk1, and crescent can influence heart development, but that their perceived role as cardiac inducers has been neither proved nor disproved. A way to determine if these molecules directly provoke mesodermal progenitor cells to undergo cardiac commitment and/or differentiation would be to inhibit the expression or functional activities of Wnt11, Dkk1, and crescent specifically. One of the difficulties in carrying out those proposed experiments is that functional redundancies among Wnt11, Dkk1, and crescent in regulating cardiogenesis may require the down-regulation of all three molecules in order to suppress heart formation. This potential predicament may explain why mice with a targeted deletion of either the Wnt11 or Dkk1 genes develop normal beating hearts[103,104]. However, another aspect to the putative cardiogenic activities of these molecules — the issue of whether antagonism of canonical Wnt signaling initiates cardiogenesis — can be more readily addressed.

In the literature, Dkk1 and crescent have been defined strictly as canonical Wnt inhibitors[98,105,106,107]. Wnt11, which signals primarily through noncanonical Wnt pathways, has also been characterized as a dominant-negative inhibitor of canonical Wnts[65,68]. The functional designations of these molecules have formed the basis of the hypothesis that suppression of canonical Wnt activity in the early mesoderm is a prerequisite for cardiac tissue formation. Specifically, it has been proposed that heart formation is dependent on the inhibition of Wnt3A and Wnt8A[34,35], which are both canonical Wnts exhibited in tissue adjacent to the precardiac mesoderm of the frog, chick, and mouse embryo[106,108,109,110,111,112]. However, published experimental data is, at best, ambiguous in support of this hypothesis.

In experiments performed with the chick embryo, canonical Wnt signaling was able to perturb cardiac development, but only when this pathway was ectopically activated during a narrow developmental window[113]. Injection of Wnt3A protein or either of the GSK3 inhibitors LiCl and SB415286 at the beginning of gastrulation (early HH stage 3) produced chick embryos with an abnormal primitive streak and no cardiac tissue. Injections at mid to late HH stage 3, and early to mid HH stage 4, promoted cardia bifida and looping defects, respectively, without any apparent inhibition in the formation of myocardial tissue. Ectopic activation of canonical Wnt signaling at late HH stage 4 to 5 yielded no ill effects on the development of the heart. Thus, suppression of cardiac tissue formation only occurred when canonical Wnt signaling was activated prior to the time when a definitive mesoderm layer is established, and in context with a larger disturbance in gastrulation[113].

The direct treatment of precardiac anterior lateral mesoderm (ALM) has generated equivocal results on the effect of canonical Wnt signaling on heart formation. Infection of chick ALM explants with virus producing either Wnt3A or Wnt8 down-regulated the expression of various myocardial marker genes, as assayed by RT-PCR[34]. These results would have been strengthened by including corresponding images of the treated and nontreated explants, as immunostained tissue gives a better sense of the how the overall tissue environment is affected by the treatments. Our own studies (Fig. 6) were not able to replicate these findings, as there was no observable negative effect on the normal cardiac differentiation of explanted HH stage 5 chick anterior lateral mesendoderm in response to treatments (Wnt1, LiCl, or SB415286) that activate canonical Wnt signaling. One explanation for the discrepancy may be the distinct culture conditions employed in these two sets of experiments. The former experiments used the mesoderm layer only in cultures containing high concentrations of both BMP4 and chick embryo extract. In contrast, our explants were cultured in minimal media, but contained both the mesoderm and underlying endoderm.

The other major evidence used to support the canonical Wnt cardiac inhibition hypothesis was the report that the preferential ablation of the β-catenin gene in the developing mouse endoderm produced embryos with multiple cardiac structures[114]. One interpretation of this outcome is that the loss of β-catenin expression may have specifically prompted noncardiac progenitors to form cardiac tissue. There is, however, an alternative diagnosis for these mice. Since these genetically altered embryos suffered from extreme abnormalities, severe alterations in tissue movements may have led to the maldistribution of organ rudiments, such as the heart. This latter interpretation may have support from studies indicating that the inhibition of canonical Wnt signaling does not promote, but suppresses, the formation of mesodermal tissues[115]. Moreover, Wnt proteins are well known for their importance in regulating the distributions and movements of tissues within the developing embryo[64,116,117].

Another issue pertinent to the discussion of Wnt signaling and cardiac induction concerns the functional attributes of Dkk1 and crescent. Because these molecules were initially characterized as canonical Wnt inhibitors, this has been accepted as their sole function. But that may not be a completely accurate depiction, as both proteins display functional activities inconsistent with canonical Wnt inhibition[62,118,119]. For example, injection of Dkk1 RNA rescues ventralized UV-treated frog embryos[80], which is a property Dkk1 shares with canonical Wnts[120]. Experimental evidence indicates

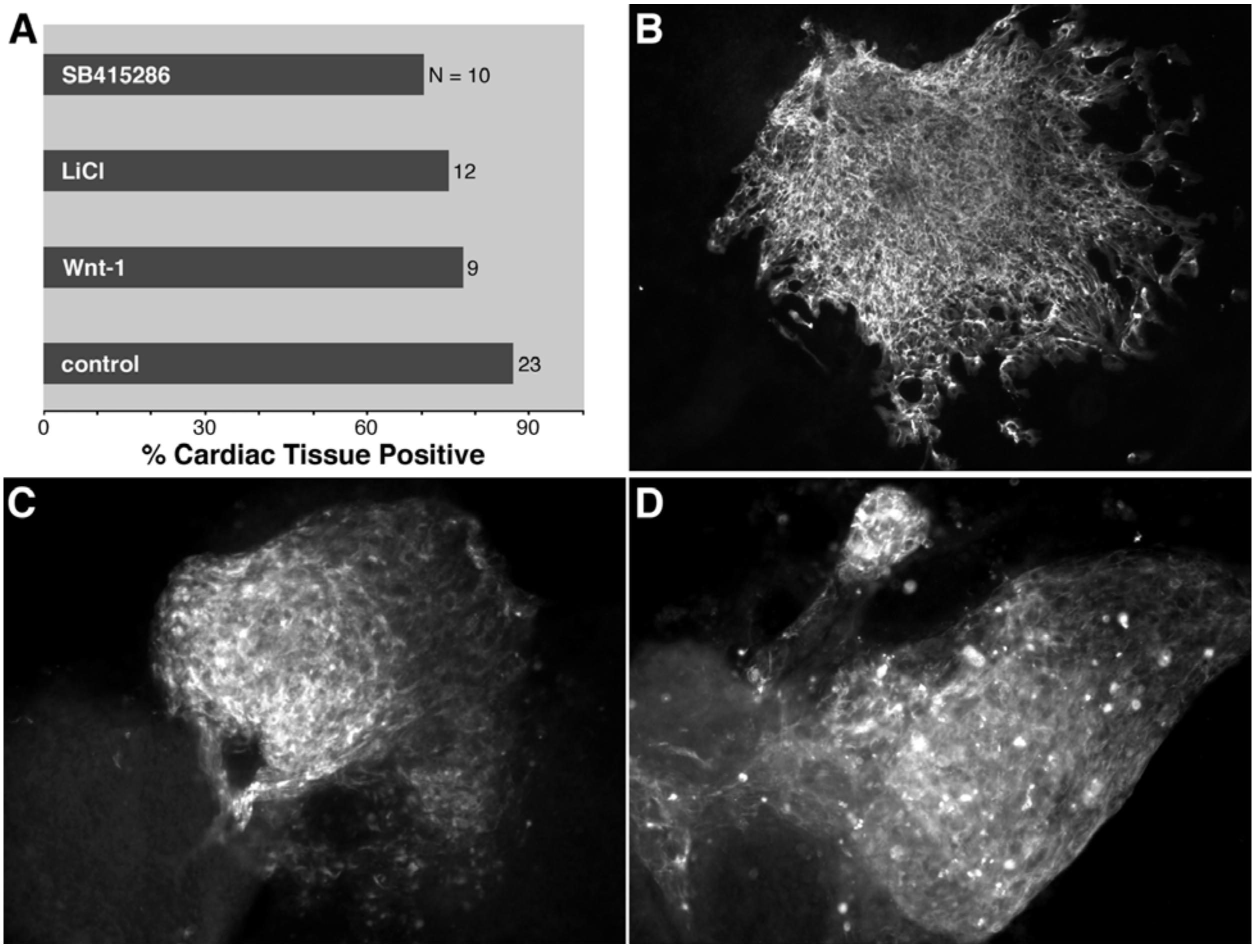

**FIGURE 6.** Canonical Wnt signaling does not prevent the myocardial differentiation of precardiac mesoderm. Explants of anterior lateral mesendoderm (ALM) were harvested from HH stage 5 chick embryos and cultured in serum-free conditions for 2 days as previously described[86,101], in the presence of control conditioned media, conditioned media from Wnt1-secreting cells, 20 m*M* LiCl, or the selective GSK3 inhibitor SB415286 (25 µ*M*). Cultures were assessed for the formation of contractile tissue and then subsequently immunostained for sarcomeric myosin or titin to confirm the cardiac tissue phenotype of the explants. (A) Scoring of cardiac tissue formation within HH stage 5 ALM explants. The total number of explants examined for each group is listed to the right of each bar. (B–D) Representative explants, treated with Wnt1, LiCl, or SB415286, respectively, and stained for (B) sarcomeric myosin or (C,D) titin after 48 h of culture. The dosages of LiCl or SB415286 used in these experiments were based on functionally optimized concentrations established in multiple published studies[137,138,139]. Wnt1-secreting cells were produced using protocols similar to that described previously for the ectopic expression of Wnt5a and Wnt11[140,141]. Briefly, a full-length mouse Wnt1 cDNA was inserted immediately downstream of the cytomegalovirus promoter in the eukaryotic expression vector pcDNA3 (Invitrogen, Carlsbad, CA), which was transfected into QCE6 cells by means of LipofectAMINE (Invitrogen). Stable transfectant cells were isolated with cloning rings following their selection by their resistance to neomycin. Wnt1-secreting clones were subsequently identified by immunoblotting using a monoclonal antibody specific to Wnt1 protein (Upstate, Charlottesville, VA).

that sFRPs, such as crescent, may have the ability to potentiate canonical Wnt responses[74,76]. Crescent also exhibits functional activities unique among sFRP proteins[77,119]. Moreover, injections of Dkk1 or crescent (or Wnt11) RNA elicit signal transduction events that have been ascribed to noncanonical Wnt pathways[62]. A further point concerns sFRP3, which is the most definitive and reliable canonical Wnt inhibitor yet characterized[121,122,123]. If inhibition of canonical Wnts is the sole story of how Dkk1 and crescent promote cardiogenesis, then sFRP3 should be a great cardiac inducer. However, sFRP3 has shown little ability to promote cardiac tissue formation[35]. These observations are not made to dismiss the potential importance of canonical Wnt inhibition for regulating cardiogenesis. Instead, the purpose here is to point out that a characterization of Dkk1 and crescent simply as Wnt inhibitors may not do justice to their full range of activities, which could lead to a less than complete understanding of their role in promoting heart formation.

## SPECULATIONS ON THE ROLE OF WNT SIGNALING IN THE FORMATION OF THE HEART

The intention of this essay is to lay out the many unresolved issues concerning the role of Wnt signal transduction in heart development. Two major elements of the current paradigm of cardiac development are that (a) antagonism of canonical Wnt signaling initiates cardiogenesis and (b) Wnt11, Dkk1, and crescent act as inducers of heart formation. As presented in the discussion presented above, a survey of the published literature indicates that (a) the importance of canonical Wnt inhibition is unproven and (b) the influence of the Wnt11, Dkk1, and crescent has not been demonstrated to be inductive. It is also unclear whether the activities of these three molecules in influencing cardiac development are equivalent, either in regard to their roles as canonical Wnt inhibitors or as promoters of noncanonical Wnt signal transduction.

If regulated Wnt signaling does not play an inductive role in the formation of the heart, what might its role be in regulating cardiac development? Many studies have established the importance of Wnt activity in regulating epithelial-mesenchymal transformations, cell migration, tissue epithelialization, tissue polarity, and organ segmentation[124,125,126,127,128,129,130,131]. In addition, Wnts are crucial for controlling major tissue movements in the embryo, such as convergent/extension movements in the early zebrafish and *Xenopus* embryo[64,116,117], and the midline convergence of organ primordia including the bilateral cardiogenic fields[132]. Crescent may exhibit similar properties, as its ectopic expression causes cyclopia[77], where midline structures fail to bifurcate and spread laterally. Thus, Wnt signal transduction greatly influences basic morphological events underlying tissue and organ development.

The distributions of Wnts, Wnt receptors, and Wnt inhibitors in the developing heart[32] suggest that Wnt signaling has a significant impact on cardiac development. The complex temporal and spatial patterns of these molecules within the embryonic heart are reminiscent of the patterns observed in other organ systems, such as the brain and kidney[133,134,135,136]. In these latter cases, it is understood that Wnt signaling shapes the emerging architecture of the developing organs. Correspondingly, Wnt signaling may play similarly important roles in shaping and remodeling the heart during development.

We therefore propose that Wnt signal transduction is not involved in specifying cardiac cell fate. Instead, regulated Wnt activity effects the establishment of the mesoderm and the epithelialization of anterior lateral mesoderm, thereby impacting the subsequent formation of the heart. The multifaceted and continual expression of Wnt signaling components in the developing heart suggest that Wnt activity plays additional roles during cardiac morphogenesis. Morphological events that are candidates for the involvement of Wnt signal regulation include the movement of the bilateral precardiac mesodermal fields toward the ventral midline, fusion of the heart-forming fields to form the tubular heart, segmentation of the developing heart, extension of the trabeculae, movement of pharyngeal mesoderm into the primitive heart, seeding of endocardial cells into the cardiac cushions, the condensation of cushion cells to form valvular tissue, segregation of conduction tissue from the working myocardium, and epicardial cell migration and integration into the heart.

## ACKNOWLEDGMENTS

This work was supported by American Heart Association grant 0555522U, NIH/NHLBI grant RO1 HL073190, and NIH Grant P20 RR-016461 from the National Center for Research Resources.
